# Clinical presentation, treatment, and antimicrobial susceptibility of 155 sequential *Staphylococcus lugdunensis* infections

**DOI:** 10.1128/spectrum.02749-24

**Published:** 2025-03-10

**Authors:** Kurt D. Palumbo, Natasia F. Jacko, Michael Z. David

**Affiliations:** 1 Division of Infectious Diseases, Department of Medicine, University of Pennsylvania 183829 , Philadelphia, Pennsylvania, USA; Icahn School of Medicine at Mount Sinai, New York, New York, USA

**Keywords:** *Staphylococcus lugdunensis*, epidemiology, skin and soft tissue infection, bacteremia, antimicrobial resistance

## Abstract

*Staphylococcus lugdunensis* is known to be virulent, but there are few large-scale epidemiologic studies of this species to define types of infection, susceptibility patterns, and severity. *S. lugdunensis* isolates from any culture at four U.S. tertiary care hospitals between 1 April 2021 and 1 April 2022 were identified. For the first isolate from each subject, clinical, demographic, and outcome data were recorded. Of 291 isolates, 223 were obtained from a clinically significant infection. Of these 223 isolates, 86 (38.6%) were from monomicrobial cultures; additionally, *S. lugdunensis* was considered a true pathogen in 69/137 polymicrobial infections. Among 155 subjects with *S. lugdunensis* infections, 49.7% were female, 46.5% were black, and 41.9% were white; 51.6% of infections were community associated. The most common infection sites were skin and soft tissue (SSTI) (*n* = 98, 63.2%), urinary tract (*n* = 16, 10.3%), and sinusitis (*n* = 14, 9%). Of nine monomicrobial bloodstream infections (BSIs), two were fatal, three involved foreign bodies, and two had infective endocarditis. Greater than half of SSTIs required an invasive procedure for cure. Among 138/291 isolates from colonization or infection, tetracycline, trimethoprim-sulfamethoxazole, oxacillin, and vancomycin susceptibility rates were 94.8% (128/135), 95.9% (94/98), 84.1% (116/138), and 100% (138/138), respectively. There were similarities in types of infection comparing *S. lugdunensis* in this study and prior reports on *Staphylococcus aureus*. SSTI was the predominant *S. lugdunensis* infection type; more than 50% of SSTIs required procedural intervention. Of nine BSIs, three involved a foreign body, and there were two cases of infective endocarditis. Oxacillin resistance was identified in 16% of isolates.

**IMPORTANCE:**

In recent years, *Staphylococcus lugdunensis* has been identified with increasing frequency as a human pathogen causing a wide variety of clinical syndromes, from soft tissue infections to fatal cases of bloodstream infection. Despite this, there are few large-scale epidemiologic studies examining this highly virulent organism. Our study adds to the growing literature on this emerging pathogen by analyzing a large case series of sequential *S. lugdunensis* infections at four U.S. hospitals to define its contemporary epidemiology, including the types of infections it causes, their outcomes, treatment approaches, and antimicrobial susceptibilities. These data provide valuable insights for clinicians in diagnosing and treating patients with these often debilitating infections. The findings also improve upon our understanding of the incidence of each infection syndrome and variability in antimicrobial susceptibilities of isolates to guide the design of future studies on the genomic epidemiology of this important pathogen.

## INTRODUCTION

First recognized as a distinct species in 1988, the coagulase-negative bacterium *Staphylococcus lugdunensis* is a commensal organism of the skin microbiome (1). However, in recent years, *S. lugdunensis* has been identified with increasing frequency as a human pathogen (2–4). Unlike other coagulase-negative *Staphylococcus* species, such as *Staphylococcus epidermidis* or *Staphylococcus capitis*, *S. lugdunensis* possesses a variety of virulence factors—cytolytic toxins, lugdunin, and distinctive heme-acquisition proteins—akin to the better-known pathogenic species, *Staphylococcus aureus* (4, 5). *S. lugdunensis* has been implicated in a wide variety of infections, sometimes fatal, including infections of the skin and soft tissues (SSTIs), bloodstream (BSI), heart valves, bones, prosthetic joints, and internal organs (2–8).

Despite its growing recognition as a pathogen, there are few large-scale epidemiologic studies examining *S. lugdunensis* (9). Most available literature on the organism consists of case reports or small case series that focus on specific clinical syndromes, such as SSTIs (10–12) or bacteremia (13–16). Certain characteristics of these infections have been suggested by some of these reports, for example, that they are particularly associated with SSTIs located below the waist (10, 17, 18) and that endocarditis is relatively common and usually severe in cases of bacteremia (6–8, 15, 16, 19–22).

Given the limited literature on *S. lugdunensis*, our primary objective was to investigate the types of infections caused by this pathogen, their outcomes, their antimicrobial susceptibility patterns, and the severity of these infections during a recent 1-year period. We also aimed to compare these findings with the general patterns observed in *S. aureus* infections. To achieve this, we conducted what we believe to be the largest retrospective case series to date, analyzing 155 clinically significant, sequential *S. lugdunensis* infections across four hospitals in Philadelphia, including their emergency departments and affiliated outpatient clinics. Our study reports the clinical, demographic, and microbiologic characteristics of these infections, along with treatment outcomes and the antibiograms of *S. lugdunensis* isolates when available.

## MATERIALS AND METHODS

### Setting and study design

We conducted a retrospective, observational cohort study for all sequential *S. lugdunensis* isolates identified from any culture (whether a true infection or from a site of colonization) by the Hospital of the University of Pennsylvania (HUP) or the Pennsylvania Hospital (PAH) Clinical Microbiology Laboratories (CML) between 1 April 2021 and 1 April 2022. The HUP CML serves HUP and HUP Cedar Avenue (together 1,200 beds, 38,611 admissions, and 101,382 emergency department [ED] visits annually) and Penn Presbyterian Medical Center (PPMC; 399 beds, 17,314 admissions, and 50,059 ED visits annually). Isolates obtained from PAH (525 beds, 19,310 admissions, and 45,352 ED visits annually) were processed by the PAH CML separately from the HUP CML.

If greater than one culture was identified from a single patient, only the first (i.e., index) culture was recorded for this study. For each of the included cases, a thorough electronic medical record (EMR) review was performed by an infectious disease clinician (K.P.) to assess the likely etiology of the infections and the role of *S. lugdunensis* according to specified criteria. The determination that *S. lugdunensis* was the primary pathogen in each case was based on culture results as well as the decision to target it as a pathogen by the treating medical providers. Patients with polymicrobial cultures that included a likely pathogen in addition to *S. lugdunensis* (e.g., *S. aureus* or *Pseudomonas aeruginosa*) were excluded.

Clinical, demographic, microbiologic, and outcome data, as noted in Table S1, were recorded in a dedicated, secure internet-based database (RedCap). No children under the age of 18 years old were included in this study. If available, antimicrobial susceptibilities were abstracted for each *S. lugdunensis* isolate. All EMR abstraction was conducted by an infectious disease clinician (K.P.).

### *E. coli* and *S. aureus* isolates from the HUP and PAH CMLs

To compare the incidence of *S. lugdunensis* isolation in the HUP and PAH CMLs with other common pathogenic bacterial species, we used the method described above to identify all sequential *Escherichia coli* and *Staphylococcus aureus* isolates from unique patients during the same period. No EMR review was performed on the patients from whom these isolates were obtained.

### Definitions

The Pitt bacteremia score was used to assess illness severity in cases of bacteremia (23). Systemic inflammatory response syndrome criteria were used to assess overall acute illness severity (24). The diagnosis of infective endocarditis was determined by the modified Duke criteria (25). Criteria for defining a urinary tract infection were adapted from the 1999 Infectious Diseases Society of America’s guidelines for antimicrobial treatment of uncomplicated acute bacterial cystitis and acute pyelonephritis in women (26). Acute kidney injury was defined as an increase in serum creatinine (SCr) by at least 0.3 mg/dL within 48 h; an increase in the SCr level to at least 1.5 times the baseline level, known or presumed to have occurred within the previous 7 days; or urine volume less than 0.5 mL/kg/h for 6 h, as defined by the KDIGO guidelines (27). The origin of infection was defined as: (i) community-associated (CA) if cultured outside of the hospital or in an inpatient setting <48 h after admission without any one or more specific healthcare exposures (surgery, overnight hospital stay, nursing home stay, or hemodialysis within the previous year; or the presence of an indwelling intravascular catheter at the time of culture); (ii) healthcare-associated (HA) if cultured in an inpatient setting >48 h after admission; or (iii) community-onset healthcare-associated (HACO) if cultured outside of the hospital or in an inpatient setting <48 h after admission with one or more of the healthcare exposures previously noted. Current use of immunosuppressive drugs was defined as the use of any immunosuppressive biologic agent, any chemotherapy, or corticosteroid use for >30 days reported within the previous 6 months. A concomitant diagnosis of “cancer” was defined as diagnosis or treatment for solid or hematologic malignancy within the 6 months prior to index infection diagnosis. Definitive antimicrobial therapies, as outlined in Table S2, were noted as either (i) the only regimen of antibiotics given, (ii) the regimen of antimicrobials given for the longest time, or (iii) the regimen of antimicrobials given last for the infection, in this order of priority. The site of infection or source of bacteremia was determined by an infectious disease clinician (K.P.) based on all evidence in the EMR.

### Microbiology

All *S. lugdunensis* isolates obtained at HUP, HUP Cedar, and PPMC were isolated and identified by the HUP CML. BACTEC FX (BD, Franklin Lakes, NJ, USA) was used to incubate blood cultures and some sterile body fluids. Other sterile body fluids, urine, sinus, tissue and wound cultures were processed by standard Gram stain and culture methods. Vitek MS (BioMerieux, Durham, NC, USA) matrix-assisted laser desorption/ionization time-of-flight mass spectrometry (MALDI-TOF) was used for species identification. Antimicrobial susceptibility testing (AST) was performed on isolates from blood cultures, sterile body fluids, surgically obtained tissue, and when it was the predominant organism in urine, sinus, and wound cultures. Additional testing was performed if requested by the clinical team. Oxacillin resistance was determined using the oxacillin MIC determined by Vitek 2 GN81 AST card (BioMerieux, Durham, NC, USA) and Kirby-Bauer cefoxitin disk testing. Current CLSI M100 guidelines were used for interpretation. If there was a discrepancy in susceptibility for the oxacillin and cefoxitin testing, *mecA* polymerase chain reaction testing was then performed. *MecA* testing was developed and validated in the HUP CML (28). All *S. lugdunensis* isolates obtained at PAH were isolated and identified by the PAH CML using the same shared protocols as the HUP CML except only Vitek2 was used for species identification.

## RESULTS

**Fig 1:**
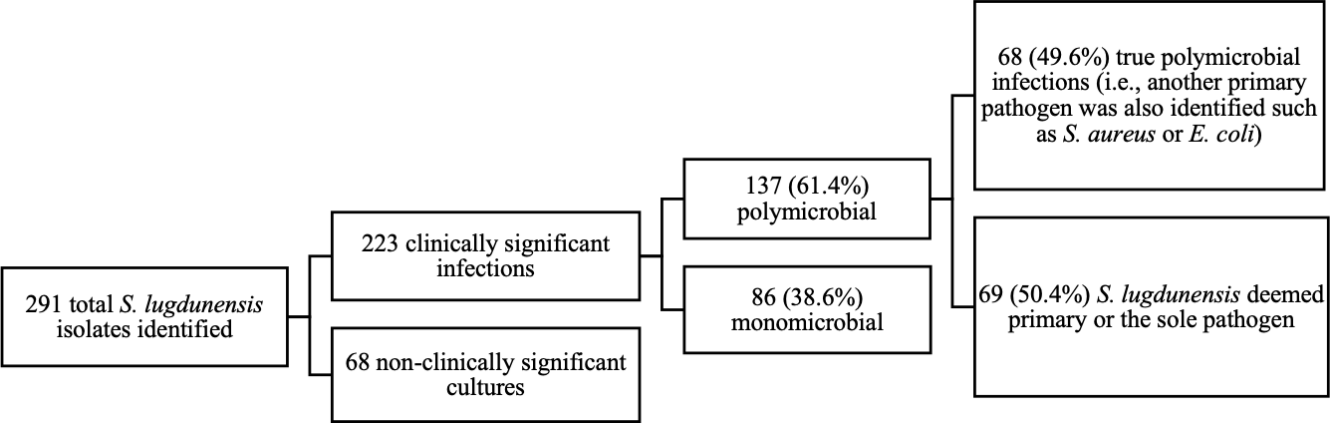
Flowchart showing derivation of cohort.

Between 1 April 2021 and 1 April 2022, 291 *S. lugdunensis* isolates from unique patients were recorded. By comparison, 7,715 *E. coli* and 3,780 *S*. *aureus* isolates were obtained from any clinical cultures in unique patients within the same time period. Derivation of the study cohort is shown in Fig. 1. Clinical characteristics and demographics of the 155 subjects with clinically significant *S. lugdunensis* infections are shown in Table 1. The median age of patients was 58 years (interquartile range [IQR] 42–70). Seventy-eight (50.3%) were male. Seventy-two (46.5%) were black. Diabetes (31.6%) and cardiovascular disease (18.7%) were the most commonly reported comorbidities. Injection drug use was reported in only two patients (1.3%). Use of immunosuppressive drugs was recorded in 13 (8.4%) patients. Greater than half of infections were epidemiologically classified as CA-*S. lugdunensis* (51.6%).

**TABLE 1:** Subject characteristics and clinical syndromes for 155 *Staphylococcus lugdunensis* infections recorded during 1 year at four U.S. academic hospitals, April 2021–April 2022

Subject characteristics	*n* = 155 (%)	Subject characteristics	*n* = 155 (%)
Age (median [IQR]), years	58 (42–70)	Intravenous drug use	2 (1.3)
Race		Immunosuppressive drug use	13 (8.4)
Black	72 (46.5)	Infection acquisition	
White	65 (41.9)	CA	80 (51.6)
Other/unknown	18 (11.6)	HA	16 (10.3)
Ethnicity		HACO	59 (38.1)
Hispanic/Latino	5 (3.2)	Infection type	
Non-Hispanic/Latino	147 (94.8)	Skin and soft tissue	98 (63.2)
Unknown	3 (1.9)	Urinary	16 (10.3)
Sex		Sinusitis	14 (9.0)
Female	77 (49.7)	Bacteremia^*b*^	9 (5.8)
Male	78 (50.3)	Osteomyelitis	6 (3.9)
Comorbidities		Joint	6 (3.9)
Cancer^*a*^	16 (10.3)	Intra-abdominal	5 (3.2)
Cardiovascular	29 (18.7)	Other	1 (0.7)
Chronic skin disease	21 (13.6)		
Diabetes	49 (31.6)		
Kidney	21 (13.6)		
Respiratory	25 (16.1)		
Transplant history	5 (3.2)		

^*a*^ Cancer was defined as diagnosis or treatment for solid or hematologic malignancy within the previous 6 months.

^*b*^ Two of the bacteremia patients had infective endocarditis as a complication.

The most common infection type caused by *S. lugdunensis* was SSTI (63.2%). Of clinically significant SSTIs (*n* = 98), 65.3% involved an anatomic site superior to the waist, and 78.6% (*n* = 77) of SSTI cultures were from an abscess or purulent wound. Skin/soft tissue swelling, redness, drainage, and pain were the most frequently endorsed symptoms. Only 8 out of 98 SSTI patients reported fever (8.2%). Invasive therapeutic procedures were performed in 65 of the 98 SSTIs, and incision and drainage were the most common (*n* = 43, 66.2%) followed by debridement (*n* = 10, 15.4%) and foreign body removal (*n* = 5, 7.7%). Of the 10 cases that required debridement, 5 required a second debridement and 1 required a third debridement to achieve source control. Doxycycline was the most often prescribed antibiotic for the definitive treatment of SSTIs (34.7%), followed by trimethoprim-sulfamethoxazole (TMP-SMX) (15.3%). These antibiotics were prescribed in some cases empirically when no AST was performed, as well as definitively when AST results were available. All SSTI patients survived and were cured of their infections.

The urinary tract (10.3%), respiratory system (9%), and the bloodstream (BSI) (5.8%) were the next most common anatomic sites of infection. All infections classified as respiratory were sinusitis, with 85.7% (*n* = 12) derived from a sinus aspirate culture; no cases of *S. lugdunensis* pneumonia were recorded. We noted just 6 cases of osteomyelitis; 4 of the 6 cases achieved surgical cure for which they received a short course of antibiotics post-operatively.

Patient and isolate characteristics and outcomes for monomicrobial *S. lugdunensis* BSI are shown in Table 2. Of reported symptoms, five of nine patients endorsed fevers and five of nine reported malaise. No BSI patients reported chills, night sweats, or rigors. Of the nine BSI cases, three involved foreign bodies (chest wall port, central venous catheter, or implantable cardioverter-defibrillator [ICD]) requiring removal. There were only two cases of *S. lugdunensis* infective endocarditis recorded, one of which was associated with an ICD lead. No BSI patients reported injection drug use within the 30 days prior to index culture. Vancomycin was prescribed to eight of nine BSI patients, and for those seven with bacteremia and no evidence of endocarditis, the mean treatment duration was 10.1 days; of the two patients with endocarditis, one received vancomycin for <1 day followed by definitive therapy with cefazolin, while the other patient received nafcillin followed by definitive therapy with cefazolin. Of the BSI patients, two died, both with Pitt Bacteremia scores at presentation higher than those who survived (12 and 4, respectively, compared with a mean of 1.6 for the seven survivors).

**TABLE 2:** Patient and isolate characteristics, and outcomes for all sequential monomicrobial *S. lugdunensis* bacteremia cases (*n* = 9) recorded at four U.S. academic hospitals, April 2021–April 2022^*a*^

Subject	Demographic characteristics	Infection acquisition	Presence of CVC at the time of positive blood culture	Suspected source of bacteremia	Comorbid conditions	Infection complications	Procedures	Pitt Bacteremia Score	Antimicrobial susceptibilities	Outcome (90 days)
Slug0013	69 yo Black male	HA	Yes	CVC	Cardiovascular, skin, kidney, respiratory	Sepsis	None	4	S: Ge, Ci, Cl, Te, Ri R: Ox	Death
Slug0043	27 yo Black female	HACO	Yes	CVC	None	Sepsis	Foreign body removal	0	S: Ge, Ci, Te, Ri R: Ox, Cl	Survived
Slug0060	51 yo White male	HACO	Yes	CVC	Cardiovascular	None	None	0	S: Ox, Ge, Ci, Cl, Te, Ri R: None	Survived
Slug0099	66 yo Black female	HACO	Yes	CVC	Cardiovascular, diabetes, kidney, respiratory	Sepsis	None	12	S: Ox, Ge, Ci, Ri R: Cl, Te	Death
Slug0103	47 yo unknown race female	HA	Yes	CVC	Respiratory	None	Foreign body removal	6	S: Ox, Ge, Ci, Te, Ri R: Cl	Survived
Slug0157	83 yo Black male	HACO	No	ICD	Cardiovascular, diabetes	Right atrial ICD lead-associated endocarditis	Foreign body removal	0	S: Ox, Ge, Ci, Te, Ri R: Cl	Survived
Slug0210	69 yo unknown race male	CA	No	Foot wound	Peripheral arterial disease, diabetes	Mitral valve endocarditis, foot osteomyelitis, CNS and pulmonary septic emboli	Valve replacement, foot debridement	2	S: Ox, Ge, Ci, Te, Ri R: None	Survived
Slug0256	61 yo Asian male	HA	Yes	CVC	Adult acute myeloid leukemia	None	None	1	S: Ox, Ge, Ci, Cl, Te, Ri R: None	Survived
Slug0261	55 yo Black male	HACO	No	Sacral wound	Cardiovascular, skin, diabetes	None	None	2	S: Ge, Ci, Cl, Te, Ri R: Ox	Survived

^*a*^ CA, community-associated; Ci, ciprofloxacin; Cl, clindamycin; CNS, central nervous system; CVC, central venous catheter; Ge, gentamicin; ICD, implanted cardiac defibrillator; Ox, oxacillin; R, resistant; Ri, rifampicin; S, susceptible; Te, tetracycline; and yo, years old.

**Fig 2:**
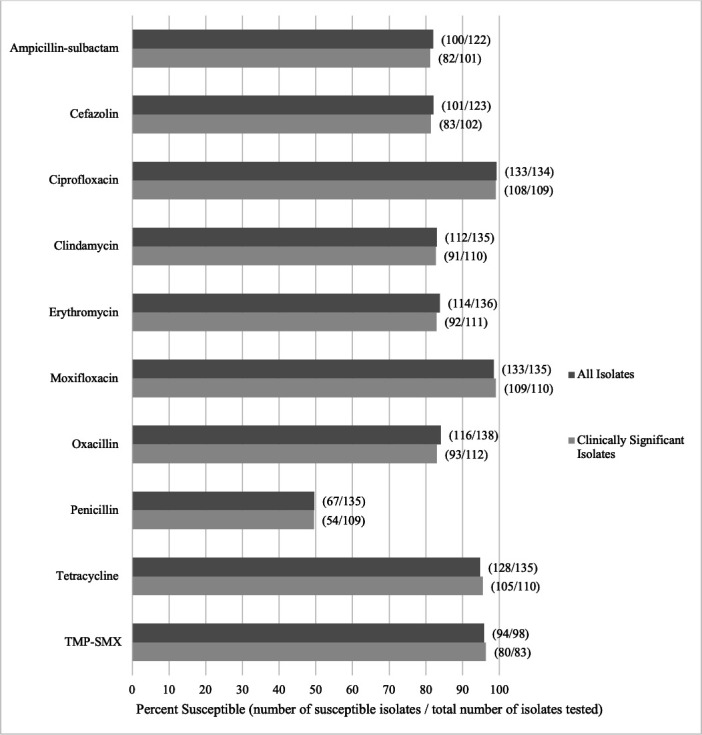
Antimicrobial susceptibilities of all 138 *S. lugdunensis* isolates with testing performed compared with the subset of isolates from clinically significant infections (*n* = 112) identified in clinical cultures at four U.S. academic hospitals, April 2021–April 2022.

AST was performed on 138 of the 291 *S*. *lugdunensis* isolates (47.4%) in the study, including some from true infections (*n* = 112) and others with colonization (*n* = 26) (Fig. 2). All isolates were susceptible to vancomycin, linezolid, tigecycline, nitrofurantoin, rifampicin, quinupristin-dalfopristin, levofloxacin, and gentamicin. Fewer than half of the tested isolates were penicillin susceptible (49.6%), and 84.1% were oxacillin susceptible. Greater than 94% were susceptible to tetracycline and TMP-SMX, whereas only 83% were clindamycin susceptible.

## DISCUSSION

This study aimed to describe the contemporary clinical epidemiology of *S. lugdunensis* infections, adding to the growing literature surrounding this highly virulent organism. *S. lugdunensis* encodes a diverse array of virulence factors, including cytotoxins that damage host cells, extracellular polysaccharide and a polyglutamic acid capsule, which enable evasion of neutrophil phagocytosis, cell-wall adhesion proteins, and iron-regulated surface determinant proteins that extract iron from heme (5, 29). In contrast, *S. aureus* has a greater number of better-characterized virulence factors, including coagulase, immune evasion proteins (such as protein A), enterotoxins, and β-barrel pore-forming cytotoxins (5). Because infections caused by *S. lugdunensis* may clinically resemble those caused by *S. aureus*, clinicians may initially suspect that they are treating an *S. aureus* infection. In the pre-MALDI-TOF era, the slide coagulase test was often used to identify *S. aureus*, but a recent French study found that it was also frequently positive with clinically derived isolates of *S. lugdunensis* (30), suggesting one potential reason for the historical misidentification of *S. lugdunensis*.

The clinical presentations of *S. lugdunensis* infections are many. Like *S. aureus*, *S. lugdunensis* is a common skin commensal and primarily causes SSTIs, with 83% of infections in one Virginia study being SSTIs (31). A Danish study reported an annual incidence of 53 infections per 100,000, with skin abscesses most frequent (10). Although BSI and endocarditis are less frequent in case series of *S. lugdunensis* compared to other staphylococci, *S. lugdunensis* endocarditis is associated with higher mortality in some reports (6, 7, 15, 20, 21, 32). Prosthetic joint and bone infections constitute up to one-third of cases (33, 34), while urinary, respiratory, and intra-abdominal infections are less common and less studied, though well documented in the literature, nevertheless.

SSTIs were the most common type of *S. lugdunensis* infection treated at our medical center, accounting for nearly two-thirds of all infections. Most SSTIs (*n* = 65, 66.3%) were treated with an invasive procedure. This may reflect the fact that patients with severe SSTIs are more likely to present for medical care or that cultures are often obtained during such a procedure. Compared with similar studies of *S. aureus*, the percentage of infections that were SSTIs was similar. For example, David et al. (35) analyzed 616 methicillin-resistant *Staphylococcus aureus* (MRSA) isolates from 2004 to 2005, finding that 57.5% were associated with SSTIs. More recent data suggest a peak in MRSA SSTIs around 2007–2008, including one study that noted an overall decline in community-onset MRSA SSTIs from 2005 to 2010 (36, 37). Among *S. aureus* SSTIs in previous studies, the percentage requiring a procedure was also similar. For example, Moran et al. (38) conducted a prospective study in 2004 across a network of university-affiliated emergency departments in 11 U.S. cities analyzing MRSA SSTIs among emergency department patients and found that 66% were treated with both incision and drainage and antibiotics. We found that the anatomic distribution of SSTIs was broad, and a majority of SSTIs involved an anatomic site above the waist, contrary to what previous reports have suggested (12, 33).

In our study at four affiliated U.S. academic hospitals, we recorded only nine *S*. *lugdunensis* BSIs during 1 year, including both oxacillin-resistant (*n* = 3) and -susceptible (*n* = 6) strains. This compares with 106 MRSA BSIs recorded at the same hospitals during a recent 12-month period (2018–2019) (39). Previous studies have reported the rarity of *S. lugdunensis* bacteremia, reflected in small case series from Switzerland, the U.S., Korea, and Japan (7, 8, 21, 22, 40). Despite such low incidence, the mortality associated with *S. lugdunensis* endocarditis remains high. A 1993 case series reported 11 cases of *S. lugdunensis* endocarditis, with a mortality rate of 72.7% (41). Later studies found lower mortality rates but still noted significant severity, particularly with left-sided native valve involvement (6, 42). More recent studies from Canada and Sweden, however, identified fewer cases and reported lower mortality (20, 43). Contemporary treatment guidelines both from the U.S. and elsewhere highlight the specific pathogenicity of *S. lugdunensis* compared with other coagulase-negative staphylococci (CoNS) causing infective endocarditis (44, 45). In our study, only two cases of endocarditis were observed, with both patients surviving at least 90 days after infection.

Antimicrobial susceptibilities of *S. lugdunensis* from the present study demonstrated similar findings to those of previous reports. Of 993 *S*. *lugdunensis* isolates from the University of California-Los Angeles CML from 2008 to 2015, McHardy et al. (46) noted 52.1% penicillin, 95.3% oxacillin, 88.5% clindamycin, and 100% daptomycin, linezolid, and vancomycin susceptibility. In our study, conducted several years later, oxacillin and clindamycin susceptibility were lower (84% and 82%, respectively). At a Greek tertiary-care hospital, 55 *S*. *lugdunensis* isolates from January 2014 to July 2017 similarly had 49% penicillin, 82% oxacillin, and 100% vancomycin susceptibility (47). Several other studies have demonstrated variable resistance patterns of *S. lugdunensis* in different parts of the world (6, 28, 48, 49), underscoring the potential for geographically variable resistance, similar to the well-documented patterns observed in *S. aureus*. Whether variable susceptibility patterns reflect underlying regional strain differences is not known.

When comparing antimicrobial susceptibility, *S. lugdunensis* differs notably from other CoNS, such as *S. epidermidis*, which commonly shows a high prevalence of methicillin resistance (47, 50, 51). Unlike these species, *S. lugdunensis* retains broad susceptibility to a wide range of antimicrobials, especially oxacillin (46, 52, 53). Analyses of the *S. lugdunensis* genomes revealed low genetic diversity and limited horizontal gene transfer, which likely contributes to the slow development of antimicrobial resistance. Like other CoNS, methicillin resistance is conferred by the presence of the *mecA* gene via horizontal transfer of staphylococcal cassette chromosome *mec* elements (51).

Over the past decade, there has been a global decline in MRSA prevalence, with stable susceptibility to non-beta-lactam antibiotics, such as vancomycin and doxycycline, for both MRSA and methicillin-susceptible *Staphylococcus aureus* (MSSA) (54, 55). In the U.S., similar trends have been observed, with *S. aureus* showing high susceptibility to vancomycin, daptomycin, and TMP-SMX and lower susceptibility to erythromycin, clindamycin, and levofloxacin (56). Interestingly, our study found *S. lugdunensis* to have 100% susceptibility to levofloxacin, a higher rate of erythromycin susceptibility, and decreased susceptibility to clindamycin, more similar to *S. aureus* (46, 51, 54, 56). Overall, *S. lugdunensis* exhibits a susceptibility pattern more similar to *S. aureus* than to other CoNS, which is crucial for selecting empiric antimicrobial treatment for invasive infections.

There are limitations to our study. First, it was a retrospective, observational cohort study. It is therefore possible that our results may not be generalizable to other centers or regions. Second, despite the large size of the examined cohort when compared with other retrospective studies, we still had limited power to examine the entire spectrum of pathology associated with *S. lugdunensis*. Third, it is possible that we misclassified polymicrobial infections as not being caused by *S. lugdunensis* when another true pathogen was present, and this assumption may have caused us to underestimate the true burden of *S. lugdunensis* at our centers. The reverse also holds true, as the burden of *S. lugdunensis* may be overestimated because it is a commensal organism to the human skin microbiome; many studied infections were SSTIs where its presence might not always have indicated that it was the cause of a true infection. We attempted to overcome this limitation by performing an extensive EMR review of each subject by an experienced infectious disease clinician (K.P.) and by applying rigorous, pre-specified criteria for true infections.

Despite these limitations, our study successfully achieved its primary goal of characterizing the features of *S. lugdunensis* infections at a large center, contributing to the growing body of literature on this emerging pathogen. By examining its contemporary epidemiology, our findings provide valuable insights for both clinicians and researchers, offering a clearer understanding of the clinical role of *S. lugdunensis* and highlighting areas for future research, including investigations of its genome, virulence factors, and antimicrobial resistance. Our findings challenge several previous assumptions about *S. lugdunensis* infections; we show that SSTIs did not predominate below the waist and that the incidence of *S. lugdunensis* BSI was quite low compared with *S. aureus*.

Our findings also highlight the striking similarities between the clinical epidemiology of *S. lugdunensis* and *S. aureus*. Greater than half of sequential *S. lugdunensis* infections were community associated, and SSTIs were by far the most common type of infection. The majority of SSTIs required procedural intervention, with incision and drainage being the most common procedure. At our hospitals, *S. lugdunensis* BSI was not common, with only nine recorded cases during a year, <10% the number of annual single-patient MRSA BSI alone recorded at the same hospitals. Almost half of BSI patients required foreign body removal for cure. The vast majority of tested *S. lugdunensis* isolates were oxacillin susceptible and had antibiograms generally similar to *S. aureus*.

### Key points

- Among 155 sequential *S. lugdunensis* infections, skin and soft tissue was the most common anatomic site; >50% of these required an invasive therapeutic procedure.
- During 1 year at four large hospitals, nine *S*. *lugdunensis* bloodstream infections were recorded.
- 16% of *S. lugdunensis* were oxacillin resistant.

## SUPPLEMENTAL MATERIAL

Supplemental material spectrum.02749-24-s0001.docx Tables S1 and S2.

ASM does not own the copyrights to Supplemental Material that may be linked to, or accessed through, an article. The authors have granted ASM a non-exclusive, world-wide license to publish the Supplemental Material files. Please contact the corresponding author directly for reuse.
