# Supplementary material for: Clinical presentation, treatment, and antimicrobial susceptibility of 155 sequential Staphylococcus lugdunensis infections
Source: Microbiol Spectr. 2025 Mar 10;13(4):e02749-24. doi: 10.1128/spectrum.02749-24 (PMC11960052; doi:10.1128/spectrum.02749-24)
Supplement: Supplemental material — Tables S1 and S2. [file spectrum.02749-24-s0001.docx]

Supplemental Table 1. Clinical, demographic, and microbiological data abstracted from electronic medical record for 155 clinically significant sequential *S. lugdunensis* infections recorded at four academic hospitals in Philadelphia, April 1, 2021-April 1, 2022

| Sex | Age | Race |
| --- | --- | --- |
| Ethnicity | Co-morbid illnesses | Hospital at which care was provided |
| Site of care where culture was obtained (emergency department, outpatient clinic, or inpatient unit) | Exposures to medical care in the year prior to *S. lugdunensis* infection (i.e., hemodialysis, hospitalization, surgery, inpatient rehabilitation or nursing home stay) | Source of culture being a clinically significant infection or colonization |
| Type of infection and anatomic site | Suspected source of infection | Current injection drug use |
| Involvement of any foreign body | Date of index culture | Vital signs as close in time to index culture available |
| Presenting symptoms | Antibiotics administered and duration | Procedures performed to treat infection |
| Complications of the infection | Complications of antibiotics administered | Outcome of infection (death during hospitalization, cure at last available visit) |
| Information about the index hospital stay (if applicable) |  |  |

Supplemental Table 2. Definitive antimicrobial therapy and mean duration, by infection type, for 155 sequential *S. lugdunensis* infections recorded at four academic hospitals in Philadelphia, April 1, 2021-April 1, 2022

| Infection Type | Definitive Antimicrobial Regimen* | Mean Duration, Days |
| --- | --- | --- |
| Skin and Soft Tissue Infection (n=98) | Amoxicillin (n=9) | 8.7 |
|  | Amoxicillin and doxycycline (n=2) | 8.5 |
|  | Cefadroxil (n=4) | 11.8 |
|  | Cefazolin (n=1) | 43 |
|  | Cephalexin (n=10) | 8.9 |
|  | Cephalexin and doxycycline (n=1) | 8 |
|  | Cephalexin and topical mupirocin (n=1) | 14 (cephalexin), 311 (topical mupirocin) |
|  | Ciprofloxacin (n=1) | 15 |
|  | Clindamycin (n=6) | 11.3 |
|  | Clindamycin and topical mupirocin (n=1) | 11 |
|  | Dicloxacillin (n=1) | 7 |
|  | Doxycycline (n=29) | 12.9 |
|  | Doxycycline (n=1) | Unknown |
|  | Doxycycline and topical clindamycin (n=1) | 91 |
|  | Doxycycline and topical mupirocin (n=2) | 7 |
|  | Doxycycline and TMP-SMX (n=1) | 7 |
|  | Levofloxacin (n=1) | 10 |
|  | Piperacillin-tazobactam (n=1) | 3 |
|  | Topical erythromycin (n=1) | 497 |
|  | Topical mupirocin (n=4) | 18 |
|  | TMP-SMX (n=13) | 10.4 |
|  | TMP-SMX (n=1) | Unknown |
|  | TMP-SMX and topical mupirocin (n=1) | 10 |
|  | Vancomycin (n=4) | 26.8 |
|  | None (n=1)** | NA |
| Urinary (n=16) | Cefpodoxime (n=2) | 7 |
|  | Ceftriaxone and amoxicillin (n=1) | 14 |
|  | Cephalexin (n=1) | 7 |
|  | Nitrofurantoin (n=5) | 5.8 |
|  | Levofloxacin (n=1) | 8 |
|  | TMP-SMX (n=6) | 6.5 |
| Sinusitis (n=14) | Amoxicillin (n=5) | 14.4 |
|  | Ceftriaxone (n=1) | 42 |
|  | Ciprofloxacin (n=1) | 15 |
|  | Piperacillin-tazobactam (n=1) | 14 |
|  | TMP-SMX (n=4) | 17 |
|  | TMP-SMX and levofloxacin (n=1) | 14 |
|  | TMP-SMX and clindamycin (n=1) | 11 |
| Bacteremia (n=9) | Cefazolin (n=4) | 28.5 |
|  | Vancomycin (n=5) | 10.8 |
| Osteomyelitis (n=6) | Amoxicillin-clavulanate (n=1)*** | 6 |
|  | Cefadroxil (n=1)*** | 7 |
|  | Cefazolin and levofloxacin (n=1) | 44 (cefazolin), 43 (levofloxacin) |
|  | Cefazolin and rifampin (n=1) | 41 (cefazolin), 37 (rifampin) |
|  | Doxycycline (n=2)*** | 11 |
| Joint (n=6) | Cefadroxil (n=2) | 279 |
|  | Cefazolin (n=1) | 42 |
|  | Daptomycin (n=1) | 28 |
|  | Linezolid (n=1) | 18 |
|  | TMP-SMX (n=1) | 43 |
| Intra-abdominal (n=5) | Cefadroxil (n=1) | 71 |
|  | Cefazolin (n=1) | 44 |
|  | Levofloxacin (n=1) | 5 |
|  | TMP-SMX (n=1) | 32 |
|  | Vancomycin (n=1) | 35 |
| Other (n=1) | Doxycycline | 12 |

*Definitive antimicrobial therapy was defined as the antimicrobial regimen meeting one or more of the following criteria, in the listed order of precedence (note that some regimens met both criteria 2 and 3):

1. The ONLY regimen of antibiotics given OR

2. The regimen of antibiotics given for the LONGEST time OR

3. The regimen given LAST to treat the infection

**Never received antimicrobials; treated with incision and drainage procedure only

***Surgical cure obtained and thus the overall antibiotic duration shortened

Abbreviations: NA, not applicable; TMP-SMX, trimethoprim-sulfamethoxazole
